# Egg parasitoids of the tea green leafhopper *Empoascaonukii* (Hemiptera, Cicadellidae) in Japan, with description of a new species of *Anagrus* (Hymenoptera, Mymaridae)

**DOI:** 10.3897/zookeys.836.32634

**Published:** 2019-04-08

**Authors:** Serguei V. Triapitsyn, Tetsuya Adachi-Hagimori, Paul F. Rugman-Jones, Adema Barry, Aoba Abe, Kazunori Matsuo, Kazuro Ohno

**Affiliations:** 1 Department of Entomology, University of California, Riverside, California, USA; 2 Organization for Promotion of Tenure Track, University of Miyazaki, Miyazaki, Japan; 3 Interdisciplinary Graduate School of Agriculture and Engineering, University of Miyazaki, Miyazaki, Japan; 4 Faculty of Agriculture, University of Miyazaki, Miyazaki, Japan; 5 Faculty of Social and Cultural Studies, Kyushu University, Fukuoka, Japan

**Keywords:** *
Anagrus rugmanjonesi
*, *
Arescon enocki
*, egg parasitoid, molecular identification, *
Stethynium empoascae
*, taxonomy, tea pest

## Abstract

Fairyfly (Hymenoptera, Mymaridae) egg parasitoids of the tea green leafhopper Empoasca (Matsumurasca) onukii Matsuda (Hemiptera, Cicadellidae), an economically important pest in Asia of the tea plant, *Camelliasinensis*, were identified from specimens reared in Japan. Using a combination of genetic and morphological evidence, Anagrus (Anagrus) rugmanjonesi Triapitsyn & Adachi-Hagimori, **sp. n.**, is described and illustrated. It is shown to be different from the most similar *A.turpanicus* Triapitsyn & H*u, an* egg parasitoid of a leafhopper pest of cultivated grapes which is known from Xinjiang Uyghur Autonomous Region in China. Mitochondrial and nuclear ribosomal DNA sequence data provide clear evidence for the separation of *A.rugmanjonesi* from *A.turpanicus* and other members of the *Anagrusincarnatus* Haliday species complex. A key to females of the Japanese species of *Anagrus* Haliday is given. Two other species of Mymaridae, *Aresconenocki* (Subba Rao & Kaur) and *Stethyniumempoascae* Subba Rao, are also identified, albeit the latter one only tentatively. Both latter taxa are newly recorded from Japan, and *E.onukii* represents their new host association.

## Introduction

**Figure 1.**
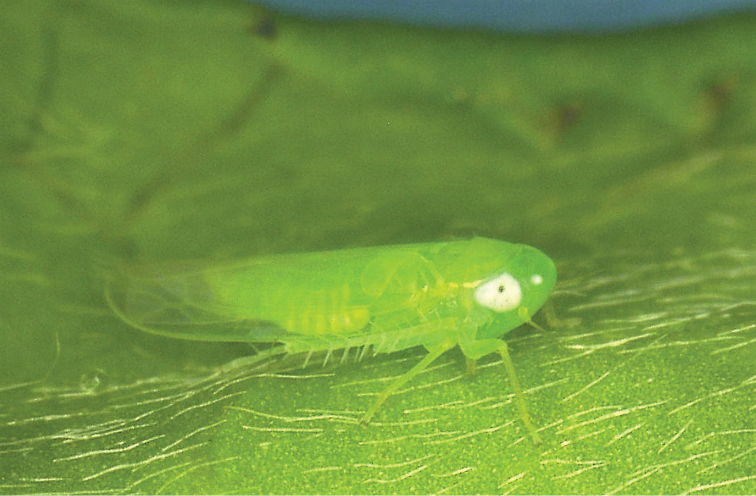
Empoasca (Matsumurasca) onukii adult feeding on a tea leaf (Miyazaki Prefecture, Kyushu Island).

The tea green leafhopper, Empoasca (Matsumurasca) onukii Matsuda (Hemiptera, Cicadellidae) (Fig. 1) is one of the major pests of tea plants in Japan and also in mainland China and Taiwan where it has been commonly misidentified as *Empoascavitis* (Göthe) and *Jacobiascaformosana* (Paoli) (or as *Empoascaformosana* Paoli), respectively (Qin et al. 2015). Adults and nymphs of *E.onukii* cause leaf vein reddening, leaf margin yellowing, leaf curling, stunted shoot growth, and leaf drop, which results in economic losses of up to 33% (Xu et al. 2005). Eggs of *E.onukii* are laid singly, embedded in the soft tissues of tea bushes, such as veins of leaves and tender stems (Takagi 1978).

In Japan, tea green leafhoppers have developed resistance to the insecticides (Ozawa et al. 2009) used intensively against this pest. Thus, development of alternative control methods is desirable. Egg parasitoids offer one potential alternative for regulating tea green leafhopper populations. Takagi (1978) found mymarid wasps (Hymenoptera, Mymaridae), known as fairyflies in English, in tea fields in Japan, and later, Ojima et al. (2010) provided data on the population dynamics of three species parasitizing eggs of *E.onukii* in tea plantations in Kochi Prefecture. However, biological control-based integrated pest management (IPM) using these egg parasitoids has not yet been established.

Unfortunately, voucher specimens of the study by Takagi (1978) could not be located, and those of Ojima et al. (2010) were lost (I. Ojima personal communication). Thus, as the first step towards establishment of biological control-based IPM using egg parasitoids, we collected fairyflies in organic tea fields and identified them both morphologically and genetically.

## Materials and methods

### Specimen collection

Tea shoots were collected from three organic tea fields in Takaoka (Takaoka, fields 4, 5, 6), Miyazaki City on October 10, 17, and 25, and in one organic tea field in Kitakata (Kita, field 1), Nobeoka City, Miyazaki Prefecture, on October 20, 2017. All tea plants belonged to variety ‘Yabukita’. In each field, 75–95 new shoots (15–20 cm length) were collected, put into plastic bags, kept in a cooler box containing ice, and transported to the Laboratory of Applied Entomology, University of Miyazaki, Miyazaki. The shoots were then transferred to two different container sets for observing either eclosion of the nymphs of tea green leafhoppers or emergence of egg parasitoid adults. The first container type consisted of plastic bottles covered with black opaque plastic film. The bottom of the bottle was cut off and replaced with the lid of a candy bottle. The latter was filled with a wetted garden sponge into which 20 tea shoots with one leaf per shoot were inserted. A transparent plastic test tube was screwed on the top of the plastic bottle. This system allowed for observation and collection of the emerged wasps from tea shoots through the test tube, as they are attracted to light. The second set of containers consisted of test tubes. A tea shoot without leaves was inserted into a small cut of wet garden sponge. The shoot inserted into wet garden sponge was put into a test tube and sealed with parafilm. The emerged wasps were collected every 24 hours and were provided with honey solution until they died naturally. The dead wasps were collected, labeled, placed in 99.5% ethanol and stored at –20 °C until they were shipped to the first author. These specimens were used both for molecular analyses and taxonomic studies (as type material of the new species described below).

### Taxonomic studies

Morphological identifications of the *Anagrus* sp., made by the first author, were based mainly on females because males of many species of *Anagrus* Haliday are often similar.

Results of the genetic analysis were key in determining the separation of the new species of *Anagrus* from *A.turpanicus* Triapitsyn & Hu from Xinjiang Uyghur Autonomous Region in China; this species is an egg parasitoid of a leafhopper pest of cultivated grapes, *Arboridiakakogawana* (Matsumura) (Hu and Triapitsyn 2016), which is the most similar based on morphology of both sexes. The genetic analysis was also useful to separate the new species from other members of the *Anagrusincarnatus* Haliday species complex (Triapitsyn et al. 2018, 2019).

For the taxonomic description of the new species, the morphological terms of Gibson (1997) and Triapitsyn (2015) were used. All measurements (as length or length: width for the wings) are given in micrometres (µm). Abbreviations used in the description and key are:

**F** funicle segment of the female antenna or flagellomere of the male antenna;

**mps** multiporous plate sensillum or sensilla on the antennal flagellar segments (= longitudinal sensillum or sensilla, or sensory ridge(s)).

Specimens from ethanol were dried using a critical point drier, then point-mounted and labeled. Selected specimens were dissected and slide-mounted in Canada balsam. Slide mounts were examined under a Zeiss Axioskop 2 plus compound microscope (Carl Zeiss Microscopy, LLC, Thornwood, New York, USA) and photographed using the Auto-Montage system (Syncroscopy, Princeton, New Jersey, USA). Photographs were retouched where necessary using Adobe Photoshop (Adobe Systems, Inc., San Jose, California, USA).

Specimens examined are deposited in the collections with the following acronyms:

**BLKU** Biosystematics Laboratory, Faculty of Social and Cultural Studies, Kyushu University, Fukuoka, Japan;

**UCRC** Entomology Research Museum, Department of Entomology, University of California, Riverside, California, USA.

### DNA extraction, amplification, and sequencing

DNA was extracted from two individual female wasps using the “HotSHOT” method of Truett et al. (2000), in a total volume of 80 µL. This non-destructive method allowed for the recovery and slide-mounting of each specimen following extraction; each slide was then labeled with the assigned P. F. Rugman-Jones’ primary molecular voucher PR number and UCRC database UCRC ENT number. For reasons described below, DNA was also extracted from one male (PR18-486) using a Chelex^100^ method described by Stouthamer et al. (1999). This specimen was destroyed by grinding and is listed below under “Other (non-type) material examined”. Two “preliminary” specimens of the same species of *Anagrus* were also subject to a destructive extraction protocol in Japan.

The polymerase chain reaction (PCR) was employed in an attempt to amplify the “barcoding” region of the mitochondrial cytochrome c oxidase subunit I gene (COI) using LCO1490 (5’-GGTCAACAAATCATAAAGATATTGG-3’) and HCO2198 (5’-TAAACTTCAGGGTGACCAAAAAATCA-3’; Folmer et al. 1994), as described in Rugman-Jones et al. (2012). This primer combination has previously proved good for use with HotSHOT-extracted specimens of *Anagrus* (e.g. Triapitsyn et al. 2018, 2019). However, in this instance, amplification of COI from the HotSHOT-extracted specimens failed for these and several alternative primer combinations (data not shown). In contrast, amplification of COI from the Chelex^100^-extracted specimen, and the two “preliminary” specimens extracted in Japan, worked fine. Reactions were performed in 25 µL volumes on a Mastercycler ep gradient S thermocycler (Eppendorf North America Inc., New York, New York, USA) and amplification was confirmed by gel electrophoresis.

In a separate PCR, the internal transcribed spacer 2 (ITS2) region of nuclear ribosomal RNA (rRNA) was amplified for all 3 specimens extracted by PRJ (HotSHOT- and Chelex^100^-extractions) using the primers, 58SF (5’-GTGAACTGCAGGACACATGAAC-3’) (Porter and Collins 1991) and ITS4 (5’-TCCTCCGCTTATTGATATGC-3’) (White et al. 1990), as described in Morse et al. (2016).

All PCR products were cleaned using a DNA Clean & Concentrator™-5 kit (Zymo Research Corporation, Irvine, California, USA) and direct sequenced in both directions at the Institute for Integrative Genome Biology, University of California at Riverside. The parity of forward and reverse reads was checked using SEQUENCHER 4.9 (Gene Codes Corporation, Ann Arbor, Michigan, USA) and priming regions were removed manually in BioEdit version 7.0.5.3 (Hall 1999). The online tool, EMBOSS Transeq (Rice et al. 2000) was used to translate the protein coding COI sequence into its amino acid chain, confirming the absence of indels and pseudogenes. All sequences were deposited in GenBank (Benson et al. 2008).

### Genetic analysis

Representative COI sequences previously obtained by Triapitsyn et al. (2018, 2019) for members of the *incarnatus* species complex (and associated out-group taxa) were combined with the current COI data. The sequence data was subsequently aligned using MAFFT version 7.050 (Katoh and Standley 2013) and the Q-INS-i algorithm with default settings. The aligned COI dataset contained 23 terminal taxa (including outgroups), 587 nucleotide positions, and no gaps. Genetic variation among our sequences was estimated by calculating uncorrected p-distances between all possible sequence pairs, using MEGA version 6 (Tamura et al. 2013). All ambiguous positions were removed for each sequence pair. A neighbor-joining (NJ) tree based on those p-distances was subsequently constructed, again using MEGA. Branch support was estimated using a bootstrap procedure with 1000 replicates.

As phylogenetic inference from ITS2 is typically problematic due to large interspecific differences that make alignment of this region difficult and somewhat ambiguous, ITS2 sequences were examined “by eye” to corroborate the status of our specimens as a single species, and to compare them with other *Anagrus* species by using a BLAST search of the NCBI database.

## Results

### Taxonomy

#### Anagrus (Anagrus) rugmanjonesi

Taxon classificationAnimaliaHymenopteraMymaridae

Triapitsyn & Adachi-Hagimori sp. n.

http://zoobank.org/26BD44A5-87B3-4AD9-968A-83B9A2B0DECC

**Figure 2.**
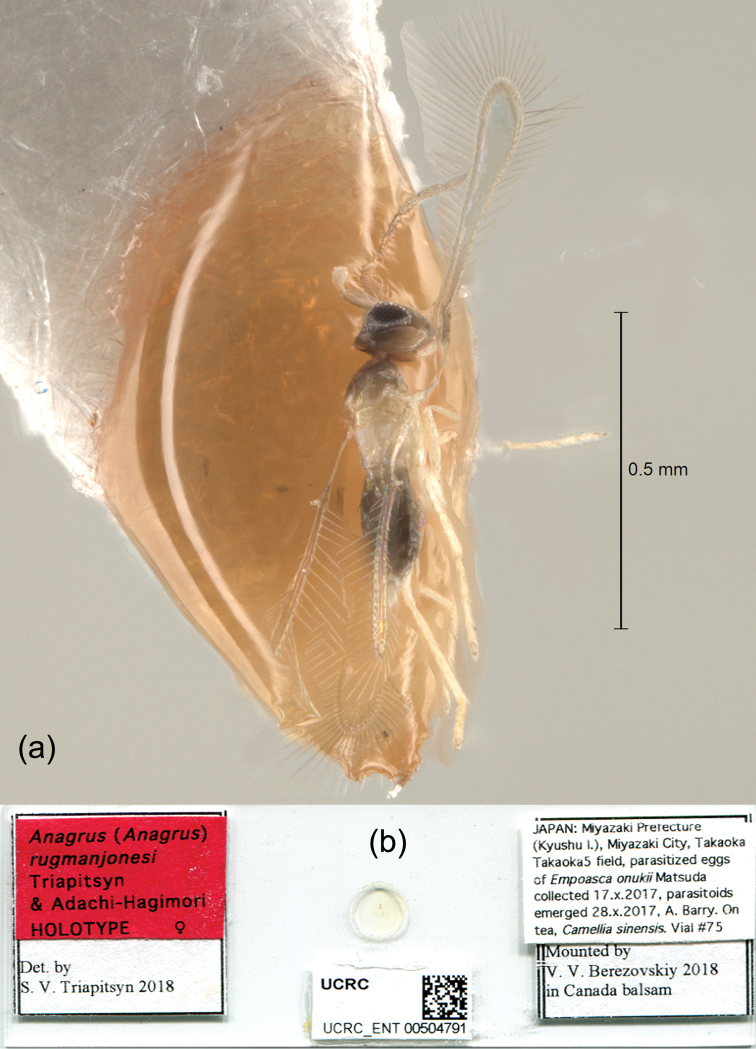
*Anagrusrugmanjonesi* sp. n. female: **a** habitus of dry-mounted specimen (paratype from Takaoka, Miyazaki City, Miyazaki Prefecture, Kyushu Island) **b** slide (holotype).

**Figure 3.**
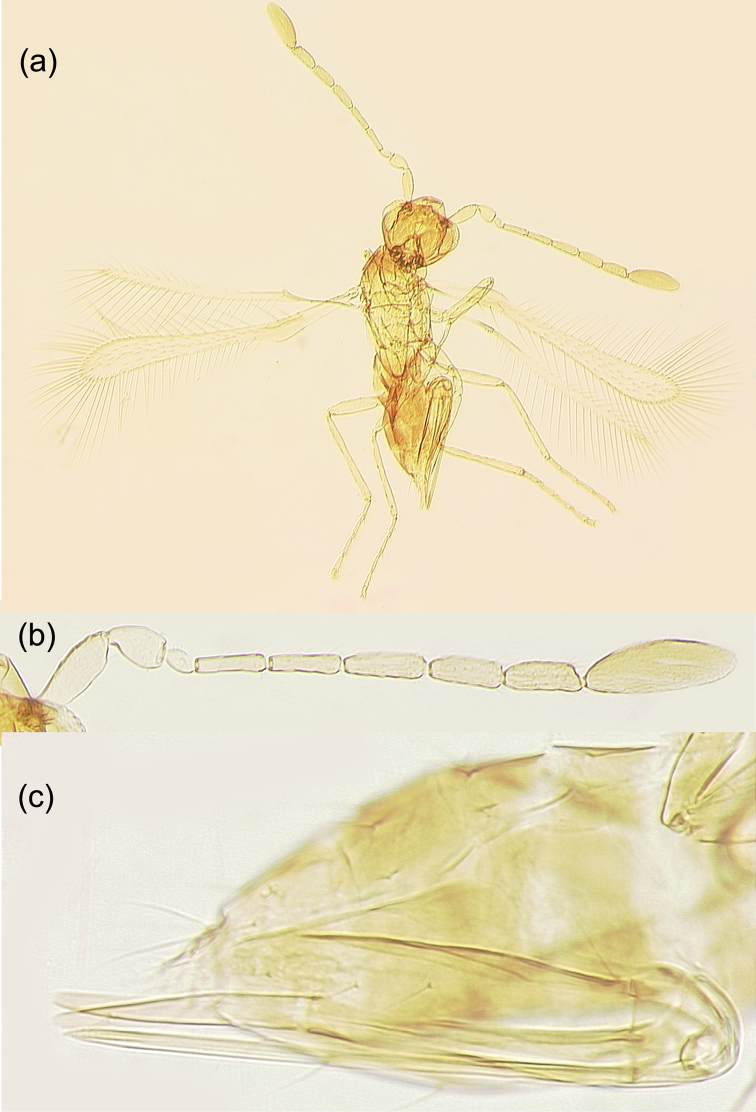
*Anagrusrugmanjonesi* sp. n. female: **a** holotype habitus **b** holotype antenna **c** metasoma (paratype from Kitakata, Nobeoka City, Miyazaki Prefecture, Kyushu Island).

**Figure 4.**
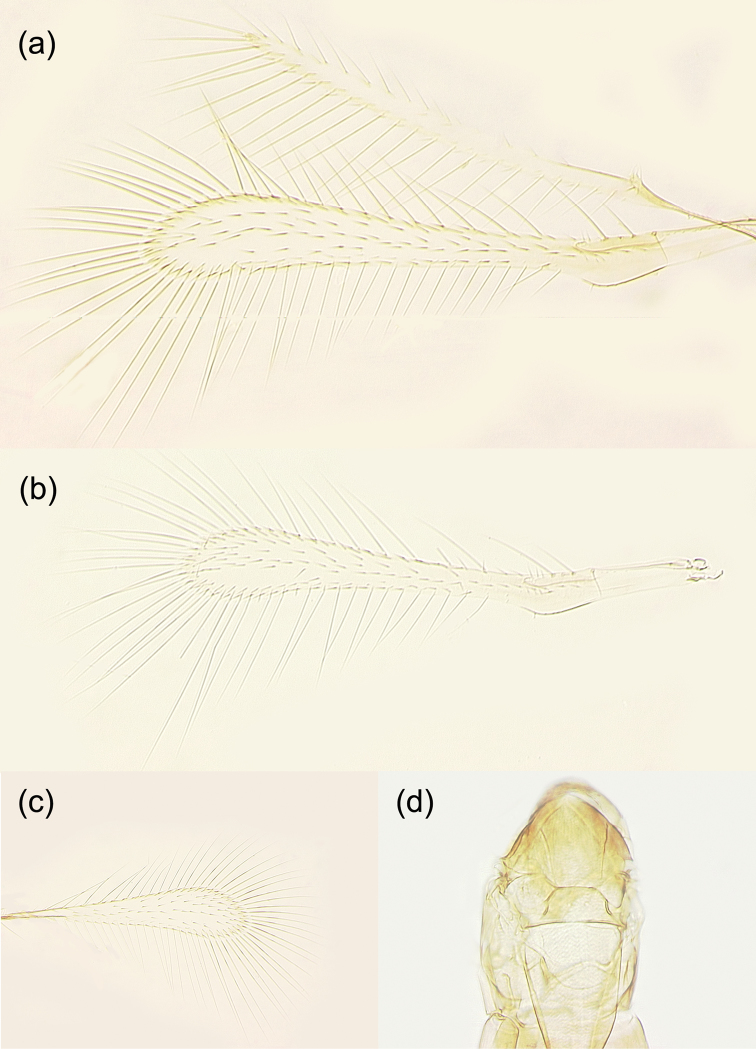
*Anagrusrugmanjonesi* sp. n. female: **a** fore and hind wings (holotype) **b** fore wing (paratype from Takaoka, Miyazaki City, Miyazaki Prefecture, Kyushu Island) **c** fore wing (paratype from Kitakata, Nobeoka City, Miyazaki Prefecture, Kyushu Island) **d** mesosoma (paratype from Takaoka).

**Figure 5.**
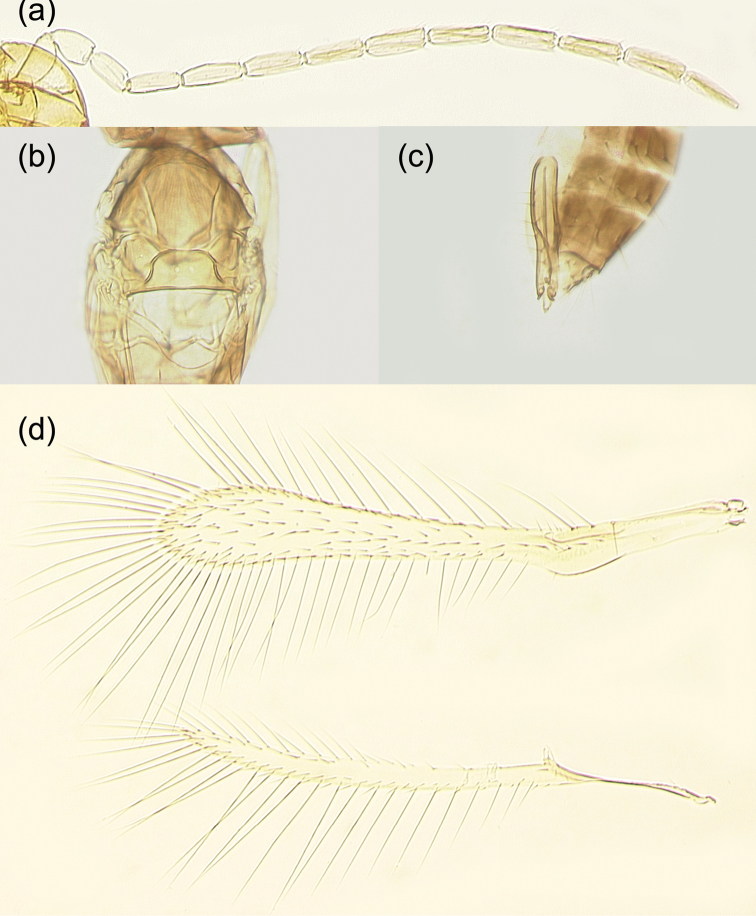
*Anagrusrugmanjonesi* sp. n. male (paratypes from Miyazaki Prefecture, Kyushu Island, Japan: **a–c**, Kitakata, Nobeoka City; d, Takaoka, Miyazaki City): **a** antenna **b** mesosoma **c** genitalia **d** fore and hind wings.

Figures 2 , 3 , 4 , 5

?Anagrus sp.: Takagi 1978: 101–102 (egg parasitoid of tea green leafhopper and its population dynamics in Japan).
Mymaridae sp. A (resembling Anagrus): Ojima et al. 2010: 38–41 (egg parasitoid of tea green leafhopper and its population dynamics in Kochi Prefecture, Shikoku Island, Japan), 43–44 (photographs).

##### Type material.

Holotype female, deposited in BLKU, on slide (Fig. 2b) labeled: 1. “JAPAN: Miyazaki Prefecture (Kyushu I.), Miyazaki City, Takaoka Takaoka5 field, parasitized eggs of *Empoascaonukii* Matsuda collected 17.x.2017, parasitoids emerged 28.x.2017, A. Barry. On tea, *Camelliasinensis*. Vial #75”; 2. “Mounted by V. V. Berezovskiy 2018 in Canada balsam”; 3. [magenta] “Anagrus (Anagrus) rugmanjonesi Triapitsyn & Adachi-Hagimori HOLOTYPE ♀”; 4. “Det. by S. V. Triapitsyn 2018”; 5. [barcode database label/unique identifier] “UCRC [bold] UCRC_ENT 00504791”. The holotype (Fig. 3a) is in good condition, complete.

Paratypes. JAPAN, Kyushu Island, Miyazaki Prefecture (from parasitized eggs of *E.onukii* on tea plant, *Camelliasinensis*): Miyazaki City, Takaoka: Takaoka 4 field, collected 17.x.2017, emerged 26.x.2017, A. Abe (vial #18) [1 female on point, BLKU (UCRC_ENT 00504790) and 1 female on slide, UCRC (molecular voucher PR18-238, UCRC_ENT 00506187)]; Takaoka 5 field, collected 17.x.2017, emerged 26.x.2017, A. Barry (vial #73) [1 female on point, UCRC (UCRC_ENT 00504789)]; Takaoka 5 field, collected 17.x.2017, emerged 31.x.2017, A. Barry (vial #71) [1 male on point, UCRC (UCRC_ENT 00504788)]; Takaoka 6 field, collected 17.x.2017, emerged 24.x.2017, A. Abe (vial #11) [1 female on slide, BLKU (UCRC_ENT 00506185)]; Takaoka 6 field, collected 17.x.2017, emerged 25.x.2017, A. Abe (vial #12) [1 male on slide, BLKU (UCRC_ENT 00506184)]. Nobeoka City, Kitakata, Kita 1 field: collected 20.x.2017, emerged 27.x.2017, A. Abe (vial #32) [1 female on slide, UCRC (molecular voucher PR18-239, UCRC_ENT 00506188)]; collected 20.x.2017, emerged 26.x.2017, A. Barry (vial #38) [1 female on slide, UCRC (UCRC_ENT 00506186)]; collected 20.x.2017, emerged 27.x.2017, A. Barry (vial #37) [1 male on slide, UCRC (UCRC_ENT 00506183)].

##### Other (non-type) material examined.

JAPAN, Kyushu Island, Miyazaki Prefecture (from parasitized eggs of *E.onukii* on tea plant, *Camelliasinensis*): Miyazaki City, Takaoka: Takaoka 4 field, collected 10.x.2017, emerged 12.x.2017, A. Abe (vial #15) [1 female in ethanol, UCRC]; Takaoka 4 field, collected 10.x.2017, emerged 19.x.2017, A. Abe (vial #17) [1 male in ethanol, UCRC]; Takaoka 4 field, collected 17.x.2017, emerged 26.x.2017, A. Abe (vial #16) [1 male in ethanol, UCRC]; Takaoka 5 field, collected 25.x.2017, emerged 31.x.2017, A. Barry (vial #72) [1 male, destroyed for DNA extraction, PR18-486]. Nobeoka City, Kitakata, Kita 1 field, collected 20.x.2017, emerged 1.xi.2017, A. Abe (vial #34) [1 female in ethanol, UCRC].

##### Diagnosis.

The new species is a member of the *incarnatus* species group of the Anagrus (Anagrus) as defined by Chiappini et al. (1996), and its *A.incarnatus* species complex, studied by Triapitsyn et al. (2018). Female antenna (Fig. 3b) with F2 not the longest funicular segment (usually F4 is or, sometimes, F6); mps on F4 (1), F5 (1 or 2), F6 (2), and clava (5); midlobe of mesoscutum without adnotaular setae (Figs 4d, 5b); fore wing disc sometimes with a distinct, but small subapical bare area (Fig. 4b) but often this bare area is either somewhat indistinct (Fig. 4a) or absent (Fig. 4c); ovipositor 2.3–2.5× length of protibia.

Morphologically, *A.rugmanjonesi* is most similar to the Palaearctic species *A.turpanicus*, to which its female specimens with a more or less distinct bare area on the fore wing disc key in Li et al. (2018). Both taxa have F2 of the female antenna not the longest funicular segment whereas in the other members of the *A.incarnatus* species complex it is the longest one (Triapitsyn 2015; Hu and Triapitsyn 2016; Triapitsyn et al. 2018). In *A.turpanicus*, however, the mesosoma is mostly yellowish brown except anterior half or so of mesoscutum is brown and frenum is yellowish white (Hu and Triapitsyn 2016), whereas in *A.rugmanjonesi* the scutellum and mesosoma (laterally, except the pronotum) are contrastingly white (Fig. 2a). Also, in *A.rugmangonesi* the clava is at most as long as the combined length of F5 and F6 whereas it is always longer than that in *A.turpanicus*. The fact that the two species also substantially differ genetically (Fig. 7) provides a good justification for their differentiation as two separate entities. In Li et al. (2018), those specimens of *A.rugmanjonesi* that lack a more or less distinct bare area on the fore wing disc key to *Anagrusnilaparvatae* Pang & Wang, a well-known egg parasitoid of rice planthoppers (Hemiptera, Delphacidae) and leafhoppers in Asia. The latter taxon was recently synonymized under *Anagrusincarnatus*, and F2 of its female antenna is always the longest funicular segment (Triapitsyn et al. 2018). A key to females of the Japanese species of *Anagrus* is provided below, as the previous key by Sahad and Hirashima (1984) is outdated.

##### Description.

Female (holotype and paratypes). Body length of dry-mounted, critical point-dried paratypes 400–460 µm, and of the slide-mounted paratypes 560–590 µm. Body (Figs 2a, 3a) mostly brown to dark brown except face, gena, and propodeum light brown and scutellum and mesosoma laterally (except pronotum) white; posterior half or so of mesoscutum and apex of gaster often light brown to off-white; scape, pedicel and F1 pale to light brown, remaining funicular segments light brown, and clava brown; legs mostly pale to light brown, wings hyaline. Antenna (Fig. 3b) with scape 2.9–3.8× as long as wide, with cross-ridges, 1.9–2.2× length of pedicel; F1 a little longer than wide, about half of pedicel length; F2 at least slightly shorter than following funicular segments, F4 usually the longest funicular segment (except sometimes F6 the longest); mps on F4 (1); F5 (1 or 2), and F6 (2); clava with 5 mps, 2.8–3.3× as long as wide, either as long as combined length of F5 and F6 or slightly shorter. Midlobe of mesoscutum without adnotaular setae (Fig. 4d). Fore wing (Fig. 4a–c) 7.0–8.0× as long as wide, longest marginal seta 2.6–2.9× maximum wing width; distal macrochaeta 1.6–1.7× length of proximal macrochaeta; disc with several rows of setae in addition to admarginal rows of setae (1 complete row originating behind apex of venation and 3 or 4 irregular rows in the broadest part of disc), sometimes leaving a distinct, but small subapical bare area at posterior margin (Fig. 4b) but often this bare area either somewhat indistinct (Fig. 4a) or absent (Fig. 4c). Hind wing (Fig. 4a) 22–24× as long as wide, longest marginal seta 6.0–7.0× maximum wing width; disc mostly bare except for an incomplete row of setae along anterior margin and a compete row of setae along posterior margin. Ovipositor (Fig. 3c) extending anteriorly almost to mesophragma in slide-mounted specimens and exserted a little beyond apex of gaster posteriorly (by 0.12–0.15× total ovipositor length). Second valvifers (= external plates of ovipositor), e.g., Chiappini et al. (1996), each with 3 setae (Fig. 3c). Ovipositor 2.3–2.5× length of protibia (2.35× in the holotype).

Measurements (µm) of the holotype (as length or length: width). Body: 535; mesosoma 190; gaster 264; ovipositor 245. Antenna: scape 70; pedicel 36; F1 18; F2 42; F3 45; F4 52; F5 45; F6 48; clava 97. Fore wing 511: 64; longest marginal seta 173. Hind wing 476: 21; longest marginal seta 127.

Male (paratypes). Body length of the slide-mounted paratypes 560–585 mm. Body color mostly as in female except entire flagellum brown. Antenna (Fig. 5a) with scape 2.4–2.7× as long as wide, F1 at least a little shorter than following flagellomeres. Fore wing 7.2–7.6× as long as wide, with or without (Fig. 5d) a more or less bare area in the broadest part. Genitalia (Fig. 5c) length 124–127 µm.

##### Etymology.

This new species is named by the first author in honor of his colleague and one of the co-authors of this communication, Paul F. Rugman-Jones, whose contributions towards determination of the identities of the nominal taxa within the *Anagrusincarnatus* species complex using molecular methods and genetic analyses have been invaluable.

##### Distribution.

Palaearctic region: Japan.

##### Host.

Cicadellidae: Empoasca (Matsumurasca) onukii Matsuda.

##### Biology.

In eggs of *E.onukii* on tea plants, *A.rugmanjonesi* was observed to develop as a solitary endoparasitoid (Ojima et al. 2010). Takagi (1978) monitored population dynamics of *A.rugmanjonesi* (as *Anagrus* sp.) in a tea plantation by using sticky suction traps. The dynamic curve indicated that *A.rugmanjonesi* was a multivoltine species and was most abundant in September. The study site of Takagi (1978) was not mentioned but is known to be the former Kanaya Town, Shizuoka Prefecture, Japan (K. Takagi personal communication), which is now part of Shimada City.

##### Comments.

The photographs of “Mymaridae sp. A” provided in Ojima et al. (2010) leave no doubt that their specimens from Kochi Prefecture, Shikoku Island belonged to both sexes of *Anagrusrugmanjonesi* n. sp.

### Key to females of the Japanese species of *Anagrus*

**Table:** 

1	Ocelli on a stemmaticum	**3**
–	Ocelli not on a stemmaticum (subgenus A. (Anagrella) Bakkendorf)	**2**
2	F2 approximately 1.5× F1 length	**Anagrus (Anagrella) brevis Chiappini & Lin**
–	F2 at least 4.0× F1 length	**Anagrus (Anagrella) hirashimai Sahad**
3	Frenum of scutellum with triangular paramedial plates widely separated from each other; metafemur short, less than 2× trochanter length, trochantellus incision almost halfway between coxa-trochanter and femur-tibia articulations (subgenus A. (Paranagrus) Perkins)	**4**
–	Frenum of scutellum with triangular paramedial plates very close to each other; metafemur long, more than 2× trochanter length, trochantellus incision about 1/3 way between coxa-trochanter and femur-tibia articulations (subgenus *A.* (*Anagrus* Haliday) [sensu stricto])	**5**
4	Ovipositor projecting beyond apex of gaster by approximately 1/3 of its total length; ovipositor length: protibia length ratio at least 3.5	**Anagrus (Paranagrus) perforator (Perkins)**
–	Ovipositor not projecting or at most slightly projecting beyond apex of gaster; ovipositor length: protibia length ratio at most 2.5	**Anagrus (Paranagrus) optabilis (Perkins)**
5	Clava with 3 mps (*atomus* species group)	**6**
–	Clava with 5 mps (*incarnatus* species group)	**7**
6	Fore wing length: width ratio > 10	**Anagrus (Anagrus) frequens Perkins**
–	Fore wing length: width ratio < 8	**Anagrus (Anagrus) japonicus Sahad**
7	Midlobe of mesoscutum with adnotaular setae	**Anagrus (Anagrus) subfuscus Foerster**
–	Midlobe of mesoscutum without adnotaular setae	**8**
8	Fore wing approximately 6.3× as long as wide	**Anagrus (Anagrus) takeyanus Gordh**
–	Fore wing at least 7.0× as long as wide	**9**
9	F2 the longest funicular segment	**Anagrus (Anagrus) incarnatus Haliday**
–	F2 at least slightly shorter than following funicular segments	**Anagrus (Anagrus) rugmanjonesi Triapitsyn & Adachi-Hagimori, sp. n.**

#### Arescon enocki

Taxon classificationAnimaliaHymenopteraMymaridae

(Subba Rao & Kaur, 1959)

Neurotes enocki Subba Rao & Kaur, 1959: 233 (illustrations), 235–237, 238 (key). Type locality: Indian Agricultural Research Institute, New Delhi, National Capital Territory of Delhi, India. Holotype female on slide [National Pusa Collection, Division of Entomology, Indian Agricultural Research Institute, New Delhi, India (NPC)] (not examined).
Arescon enocki (Subba Rao & Kaur): Subba Rao 1966: 187–189 (description of the male, illustration of the female, distribution, host association); Triapitsyn and Berezovskiy 2003: 9 (compared with Aresconzenit Triapitsyn & Berezovskiy, distribution, host association); Triapitsyn 2016: 138–140 (key, taxonomic history, redescription, diagnosis, distribution, hosts, comments), 142 (illustrations).
Mymaridae sp. C: Ojima et al. 2010: 38–41 (egg parasitoid of tea green leafhopper and its population dynamics in Kochi Prefecture, Shikoku Island, Japan), 44 (photographs).

##### Distribution.

India and Pakistan (Triapitsyn 2016), as well as Japan (new record).

##### Hosts.

Cicadellidae: *Amrascabiguttula* (Ishida) [= *Amrascabiguttulabiguttula* (Shiraki)] (Subba Rao 1966 [as *Empoascadevastans* Distant]) and *Jacobiascalybica* (de Bergevin & Zanon) [= *Empoascasignata* (Haupt)] (Triapitsyn and Berezovskiy 2003 [as *Empoascalibyca* [sic] (de Bergevin & Zanon)]; Triapitsyn 2016), as well as Empoasca (Matsumurasca) onukii Matsuda (new record).

##### Comments.

This species was redescribed and illustrated by Subba Rao (1966) and Triapitsyn (2016) based on specimens from India, so it is quite easily recognizable; particularly, Triapitsyn (2016) provided habitus digital images of both sexes of this species. The photographs of “Mymaridae sp. C” provided in Ojima et al. (2010) leave no doubt that their specimens belonged to both sexes of *A.enocki*, which thus is newly recorded from the Palaearctic region, where it is definitely an Oriental fauna element in southern Japan where tea is grown. Unfortunately, as their material was lost, we are unable to further illustrate the Japanese specimens of this species.

#### Stethynium ? empoascae

Taxon classificationAnimaliaHymenopteraMymaridae

Subba Rao, 1966

Figure 6

Stethynium empoascae Subba Rao, 1966: 189, 191, plate V [the figures are mislabeled as “Lymaenonempoascae”]. Holotype female, Delhi, India [NPC] (not examined).
Stethynium triclavatum Enock: Huber 1987: 829 (synonymy).
Stethynium empoascae Subba Rao: Triapitsyn 2002: 10–11 (resurrection as a valid species, taxonomic history, diagnosis, distribution, hosts, comments).
Mymaridae sp. B (resembling Anagrus): Ojima et al. 2010: 38–41 (egg parasitoid of tea green leafhopper and its population dynamics in Kochi Prefecture, Shikoku Island, Japan), 44 (photographs).

##### Material examined.

JAPAN, Kyushu Island, Miyazaki Prefecture, Nobeoka City, Kitakata, Kita 1 field (from parasitized eggs of *E.onukii* on tea plant, *Camelliasinensis*): collected 20.x.2017, emerged 27.x.2017, A. Abe [1 female, UCRC]; collected 20.x.2017, emerged 23.x.2017, A. Barry [1 female, UCRC]; collected 20.x.2017, emerged 30.x.2017, A. Barry [2 females, BLKU, UCRC]; collected 20.x.2017, emerged 31.x.2017, A. Barry [1 female, UCRC]; collected 20.x.2017, emerged 1.xi.2017, A. Abe [1 female, UCRC].

##### Distribution.

Australia (Queensland) (Triapitsyn 2002), India (Subba Rao 1966), and Japan (new record).

##### Hosts.

Cicadellidae: *Amrascabiguttula* (Ishida), *Austroascaalfalfae* (Evans), ?*Empoasca* sp., and *Jacobiascalybica* (de Bergevin & Zanon) (Triapitsyn 2002), as well as Empoasca (Matsumurasca) onukii Matsuda (new record).

##### Comments.

The photographs of “Mymaridae sp. B” provided in Ojima et al. (2010) leave no doubt that their specimens belonged to both sexes of a *Stethynium* sp., which almost certainly were conspecific with ours from the same genus.

**Figure 6.**
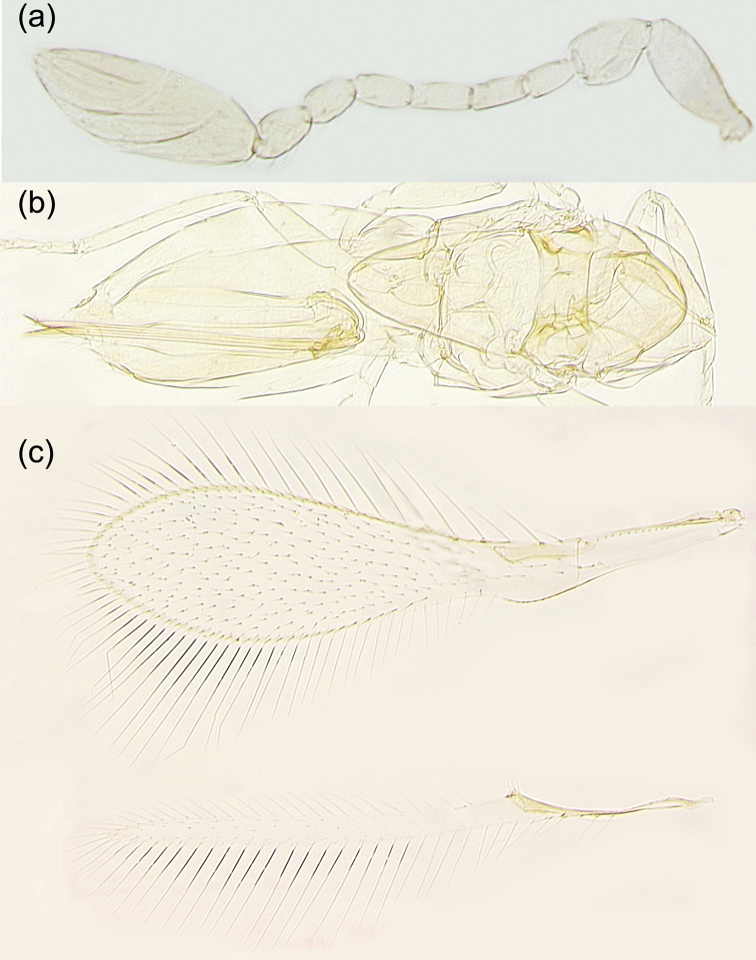
*Stethyniumempoascae* female (from Kitakata, Nobeoka City, Miyazaki Prefecture, Kyushu Island): **a** antenna **b** mesosoma and metasoma **c** fore and hind wings.

As discussed by Triapitsyn (2002), *S.empoascae* is extremely similar morphologically to usually lighter-colored specimens of *Stethyniumtriclavatum* Enock to the extent that it may be impossible to distinguish them in some countries (like China, Egypt, India, Japan, Nepal, Pakistan, etc.) where both species can potentially occur. Yet, females of *S.empoascae* from Australia and India, which could be a different, more subtropical and tropical species, seem to be slightly different from the majority of the European and North American specimens of *S.triclavatum*, which supposedly occurs in the countries with a more temperate climate (Triapitsyn 2002). Ultimately, molecular studies comparing freshly preserved specimens from Australia, Europe, India, Japan, and North America (now lacking) would need to be conducted to confirm separation of these two nominal species or, otherwise, provide genetic evidence of their possible conspecificity. At this point, however, we can only tentatively assign our specimens to *S.empoascae* based on some of the very minor morphological features mentioned in Triapitsyn (2002) as well as the fact that they were collected in Japan on the two islands with a subtropical climate. To facilitate recognition of this species, we provide illustrations of its female antenna (Fig. 6a), mesosoma and metasoma (Fig. 6b), and a pair of wings (Fig. 6c).

### Molecular analyses

**Figure 7.**
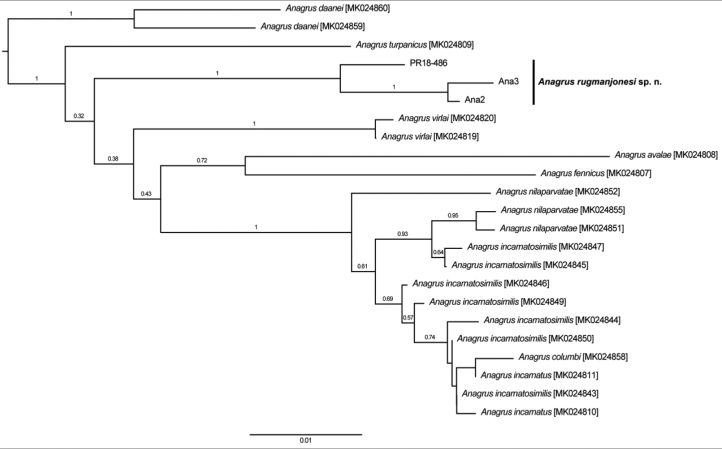
Relationship of *Anagrusrugmanjonesi* sp. n. with other member of the *A.incarnatus* species complex, based on a 587 bp fragment of COI. Optimal unrooted NJ tree with the sum of branch length = 0.27005784. The percentage of replicate trees in which the associated taxa clustered together in the bootstrap test (1000 replicates) are shown next to the branches and the tree is drawn to scale, with branch lengths indicating uncorrected p-distance.

Sequences of the COI gene provided strong evidence that *A.rugmanjonesi* is distinct from *A.turpanicus* and other members of the *A.incarnatus* species complex. Three COI haplotypes were identified for *A.rugmanjonesi*, with maximum 1.9% divergence (based on uncorrected p-distances) among those haplotypes (GenBank accessions MK544853-MK544855; Fig. 7). All substitutions were synonymous. In turn, *A.rugmanjonesi* was at least 5.1% divergent from all other accepted species in the *incarnatus* species group, with *A.turpanicus* being the most similar (Fig. 7). The exact relationship between *A.rugmanjonesi* and *A.turpanicus* (and indeed other species) was largely unresolved, with weak branch support towards the base of the NJ tree (Fig. 7).

The ITS2 sequences of the three PRJ-extracted specimens were each 603 bp long and identical, confirming them as a single species (GenBank accessions MK564750-MK564752). A BLAST search revealed no match with anything currently in the GenBank database; the closest accessions again belonging to *Anagrusturpanicus* (MK024909-MK024911). A MAFFT alignment of our sequences with those of *A.turpanicus* resulted in a matrix with many substitutions and several sizable indels, resulting in a difference in length of approximately 40 bp.

## Discussion

This study further confirms the effectiveness of simple molecular techniques for separating morphologically similar species, in this case *A.rugmanjonesi*, *A.turpanicus*, and other members of the *A.incarnatus* species complex (Fig. 7). Description of *A.rugmanjonesi* would be difficult based solely on morphology because of its close morphological similarity to *A.turpanicus*. The convincing molecular data confirms that two species are involved. Their separation also makes sense from the habitat point of view, the latter species being an egg parasitoid of a leafhopper pest of grapevines in a very hot and dry environment of a desert oasis in Xinjiang Uyghur Autonomous Region of China. Morphologically, however, they are clearly two sister species, and that is also corroborated by the genetic data presented herein.

Lin (1981) reported one undescribed species of *Megaphragma* Timberlake (Hymenoptera, Trichogrammatidae) as an egg parasitoid of *E.onukii* (as *Empoascaformosana* Paoli) on tea plant in Taiwan, but that record was obviously erroneous, likely due to an inadequate rearing method, because members of this genus are known to be egg parasitoids of thrips (Thysanoptera).

## Supplementary Material

XML Treatment for Anagrus (Anagrus) rugmanjonesi

XML Treatment for Arescon enocki

XML Treatment for Stethynium ? empoascae
